# Understanding the relapse process: exploring Iranian women’s substance use experiences

**DOI:** 10.1186/s13011-019-0216-3

**Published:** 2019-06-18

**Authors:** Maryam Khazaee-Pool, Tahereh Pashaei, Roghayeh Nouri, Parvaneh Taymoori, Koen Ponnet

**Affiliations:** 1 Department of Public Health, School of Health, Mazandaran University of Medical Sciences, Sari, Iran; 2 Health Sciences Research Center, Addiction Research Institutes, Mazandaran University of Medical Sciences, Sari, Iran; 3 Environmental Health Research Center, Research Institute for Health Development, Kurdistan University of Medical Sciences, Sanandaj, Iran; 4 Department of public health,Faculty of Health, Kurdistan University of Medical Sciences, Sanandaj, Iran; 5 Department of Communication Sciences, imec-mict-Ghent University, Ghent, Belgium

**Keywords:** Iranian women, Relapse, Drug use, Qualitative study

## Abstract

**Background:**

Relapse is one of the main challenges that must be tackled in the drug addiction treatment. Different factors contribute to the relapse process but it remains unclear how relapse occursin women. Describing the relapse phenomenon in women might be of interest to practitioners and academics. The aim of this study was to explore the relapse experiences of Iranian women with a substance use disorder.

**Methods:**

Qualitative in-depth interviews were conducted with women with a substance use disorder. The interviews contained open-ended questions regarding relapse experiences during previous treatment. Interviews were digitally recorded. Data were analyzed using the content analysis method.

**Results:**

In total, 20 women who use drugs took part in the study. The mean age of the women was 34.57 (age range = 9.6 years), and the minimum age of participants was 23 years. The following five main themes were explored: socioeconomic backgrounds, physical complications of drug withdrawal, psychological burden of drug withdrawal, family atmosphere, and cultural factors. The findings highlighted the different treatment needs in women with a substance use disorder.

**Conclusions:**

Based on the interviews, it seems necessary to develop female-specific comprehensive treatment programs by putting more emphasis on pain treatment intervention, relapse prevention, the diagnosis and treatment of mental disorders, couples counseling, and financial support. Furthermore, policymakers should be committed to providing a nonjudgmental social environment to remove or reduce stigma of women with drug use problems.

## Background

Substance abuse is a major national concern in Iran; according to the Iranian Drug Control Headquarters (2018), 2.8 million Iranians are addicted to illicit drugs [1]. Some studies suggest that 9.6% of Iranian addicts are women [2, 3]. Also, according to the Iranian Ministry of Health and Medical Education, the male/female ratio of substance use disorder is 8:1 [4].

Studies have revealed that the percentage of women who use drugs in addiction treatment is very low [5, 6]. Women often encounter various personal, social, cultural, and structural barriers to entering treatment, such as a lack of treatment for pregnant women, low level of education, psychiatric disorders, history of trauma, stigma, discrimination, shame, poverty, child care duties, low social support, lack of services in accordance with women’s needs, high treatment cost, and rigid treatment schedules [5, 7–9]. Dolan indicated that only 20% of Iranian women who are dependent on illicit drugs receive addiction treatment services [9]. In another study, this rate was even lower: 10% [2]. In Iran, most clients who seek treatment are men, so developing a treatment regimen that is tailored to women is not a priority. However, a male-orientated treatment program might ignore some of the needs of female drug users [10]. The outcomes are not promising because addiction treatment services do not address women’s needs [11]. Relapse is one of the main challenges that need to be tackled in drug addiction treatment and is caused by a combination of individual, physiological, situational, and sociocultural factors [12–14]. In a country with a high burden of addiction—like Iran—increasing retention in drug treatment, particularly the retention of women with a substance use disorder, could also lead to reduced HIV risk [8, 15]. Furthermore, the results of this study might be useful for identifying high-risk situations leading to drug use and relapse among women. This will help therapists design appropriate relapse prevention interventions.

Using a qualitative design, the aim of this study was to explore the relapse experiences of Iranian women with a drug use disorder.

## Methods

### Participants

This study was conducted in Sanandaj city. Sanandaj is the capital of the Kurdistan province. It is located in northwestern Iran and is the second largest Kurdish city [17]. From March to July 2017, we conducted 23 in-depth qualitative interviews with women with a substance use disorder. Three interviews were excluded because the participants did not complete the interview due to personal reasons. Fourteen women were undergoing methadone maintenance treatment (MMT) during the interview. The participants were randomly selected from five MMT clinics (two patients per governmental clinic) and one MMT clinic in the central prison in Sanandaj city (four patients). Six additional women were recruited from a medium-term residential center that provides treatment services that typically last less than a month and that are based on self-help and a 12-step recovery program. The inclusion criteria in this study were that women (a) were using illicit drugs, (b) had been diagnosed as drug dependent, (c) had no mental health problems, and (d) had past relapse experiences.

In this study, relapse was defined as self-reported drug use after previous treatment and the return to drug use. To gain rich and varied data, we applied a purposive sampling technique so that participants with varying socioeconomic backgrounds, various types of drug experiences, different age groups, and varying marital statuses are represented in the study. Sampling continued until data saturation was reached [18]. An institutional review board of the Kurdistan University of Medical Sciences approved this study. [IR.14/23312]. Written informed consent was obtained from all participants.

#### Data collection

The data were collected using in-depth interviews. The corresponding author conducted all the interviews. The interviewer (TP) has received professional training from qualitative method experts in several workshop and is experienced in designing and conducting quality in-depth interviews and focus group discussions (including proper guide development and interviewing/moderating skills and techniques) with vulnerable groups, like women with a substance use disorder.

To accumulate more detailed information, semi-structured, open-ended interviews were conducted with participants. The interview guide was developed based on the literature [16–18] and piloted with 3 interviews. The interviewer followed a guide that aimed to encourage participants to explore and discuss their views and experiences about relapse regarding substance use. The interview guide included (1) the demographic characteristics of participants; (2) the history of the illicit drug use; (3) the history of addiction treatment attempts and the number and kind of treatment, such as MMT, buprenorphine, or opium tincture; (4) the history of the number of relapses to illicit drug use; (5) the experiences of relapse; (6) the identifying factors related to relapse to illicit drug use; and (7) the reasons of returning to illicit drug use. The interview guide is presented in Appendix A.

We focused on each of the expressed experiences of returning to illicit drug use in replies to the following open-ended and non-directive questions: “How many times you have been in treatment before?” “What do you most like or dislike?” “Did it impact your using behavior?” “How?” “Would you please explain more?” After each question, participants were encouraged to elaborate on what they had said. For example, they were asked, “What do you mean?” to obtain a deeper understanding of their behaviors. The interviews were conducted at MMT centers and at the central prison. Interview sessions were held in private situations, and participants were told that they could end the interview at any time. Each interview lasted between 90 and 120 min. All interviews were digitally audio recorded and then transcribed by the researchers.

#### Data analysis

This study was inspired by Graneheim and Lundman’s (2004) approach to qualitative content analysis, which focuses on manifest and latent data content. Analysis of what the content displays deals with the content features and refers to the visible, obvious parts, referred to as the obvious content. In contrast, analysis of what the content discusses deals with the connection part and contains an explanation of the main concept of the text, discussed as the hidden content. Both obvious and hidden content deal with explanation, but the explanations differ in depth and level of abstraction [15]. Data analysis was performed during data collection. More specifically, data analysis began with the data-gathering process. Each interview was transcribed and analyzed before the next interview.

The analysis process was carried out in the following stages. First, we reached an understanding of the data by reading and rereading the transcribed text. Second, we extracted the unit meanings. Third, data analysis was continued by applying line-by-line coding (i.e., labeling of a meaning unit), and codes were generated through frequent discussions between investigators. Fourth, the meaning units (i.e., the constellation of words or statements that relate to the same central meaning) were labeled with a code based on their content. Finally, based on codes with similar meanings, themes and subthemes were generated. After this, all codes that were included in the aim of the study were compared, similarities were determined, and codes were sorted into categories (i.e., groups of content that share a commonality) [15, 19].

All transcripts were imported into the MAXQDA software. Overall, five key themes and 32 subthemes emerged as main factors related to relapse among Iranian women with a substance use disorder. The framework is presented in Table 2.

As a means of validation, all interview transcripts and the codes that emerged were randomly offered to some of the women, and we asked about their opinions of the meaning of each code. When conflicting opinions arose, the women’s corrective notes were used. Additionally, the interview transcriptions were made anonymous and were offered to investigators who did not participate in this study but who had experience with qualitative research as external observers. We requested that they check the coding process’s accuracy.

### Ethics

Approval for conducting the study was granted by the Ethics Committee of Kurdistan University of Medical Sciences, Sanandaj, Iran. All women were informed that their participation in this study was voluntary, that their privacy would be maintained, and that no participants’ names would be used in any publications resulting from the study. Informed written consent was gained from each woman.

## Results

In total, 20 women with a substance use disorder took part in the study. The mean age of the women was 34.57 years (age range = 9.6 years, minimum age = 23 years), and most of them (70%) were married. The study participants’ characteristics are presented in Table 1. Overall, five major themes relevant to the purpose of this study were identified: (1) physical complications of drug withdrawal, (2) psychological burden of drug withdrawal, (3) family atmosphere, (4) socioeconomic conditions, and (5) cultural factors. For more details, see.

**Table 1:** Descriptive statistics of participants (*n* = 20)

Variable	Number	%
Job		
Full time	1	5
Part time	6	30
Jobless	13	65
Marriage		
Single	2	10
Marriage	14	70
Separated or divorced	4	20
Age		
20<	3	15
20–35	11	55
35–50	6	30
Main drug		
Opium	12	60
Tramadol	4	20
Cannabis	4	20
Injection		
Yes	5	25
No	15	75
Poly substance use		
Yes	16	80
No	4	20

Table 2. The mean length of time since relapse was 53 days (range = 4–97 days). The substances mainly used by the participants included opium and opium juice (55%), amphetamine (5%), heroin (15%), crack heroin (20%), and cannabis (5%). Below, we describe the five themes.

**Table 2:** Main themes and sub themes

Themes	Subthemes
Physical complications from drug withdrawal	Weakness and lethargy
	Physical pain
	Sexual dysfunction
	Insomnia
Psychological burden from drug withdrawal	Temptation
	Craving
	Inability to bear pressure
	Coping with stress
	Feelings of frustration
	Failure
	Adaptation problems to societal norms
	Anxiety
Contributing factors from family atmosphere	Rejection by family
	Lack of family support
	Family conflicts
	Divorce
	Neglect from family
	Addiction in the family
	Spousal betrayal
	Failure to maintain romantic relationships
Socioeconomic Conditions	Interaction with people with a substance use disorder
	Lake of appropriate treatment
	Easy access to drugs
	Rejection from friends
	Unemployment
	Poverty
	Low cost of drugs
Cultural Factors	Stigmatization
	Misconceptions about recreational drug use
	Sexual disharmony and power imbalance between treatment provider and client
	Discrimination

### Physical complications from drug withdrawal: weakness and lethargy, physical pain, sexual dysfunction, and insomnia

For some participants, the physical complications of drug withdrawal during the reentry situation were related to relapse. Participants identified multiple physical problems, such as weakness and lethargy, body pain, sexual dysfunction, and insomnia, combined with limited access to care. One participant said that the biggest threats to her health after opioid withdrawal were body pain and insomnia combined with continuity of craving:

“[The major threat] to my health [after drug withdrawal]? I’m having bone pain, and I cannot bear it... My pain tolerance is low like that... I am almost tempted to reuse because of [the] pain and insomnia. I nearly went into a coma” (P7; 36 years old, divorced, high school education, an opioid user).

Respondents also believed that the use of illicit drugs improved their sexual relationships with their partners and that the sudden lack of such improved relationships was responsible for their early consumption of illicit drugs after withdrawal. For example, one woman explained,

*“For seven months after withdrawal, my husband was always unhappy about our sexual relationship... I have a lot of sexual pleasure when I use amphetamine … I started to reuse amphetamine...”* (P4; 39 years old, married, university education, an amphetamine and opioid user).

If people are dependent on a substance, withdrawal symptoms will begin when the substance is removed. For several people with a substance use disorder, it is quite difficult and inadvisable for them to try to effectively navigate a withdrawal period. For many people who use drugs, the painful physical complications of withdrawal (such as weakness, lethargy, body pain, sexual dysfunction, and insomnia) lead to continued drug use and impede the decision to start recovery.

### Psychological burden from drug withdrawal: temptation, craving, inability to bear pressure, coping with stress, feelings of frustration, failure, adaptation problems to societal norms, anxiety

Temptation and craving occurred unexpectedly. One woman with a history of heroin dependence explained her psychological difficulties, such as frustration and hopelessness, after her drug withdrawal:

“I was tempted a lot. When I got into something stimulating or when I was faced with a bad situation, I indeed didn’t know how I could help myself, *and often the motivation to use [drugs] came from nowhere*” (P2; 47 years old, divorced, primary school education, a heroin user).

Craving occurred automatically. Some participants described the desire to consume illicit drugs while they were in withdrawal.

“*I could escape the conscious desire, as long as the physical pain wasn’t that intense. There’s a tendency for drug use... I don’t know why that craving could not be omitted … The desire to use drugs is still there... When in a bad state, I want to use illicit drugs. There is no way to resist”* (P4; 39 years old, married, university education, a heroin user).

Social pressure is one of the reasons that women relapse. One woman said,

“I feel inferior. I am unemployed, [have been] rejected by my family, and feel a deep sense of loneliness. There is a lot of pressure on me. I think that I can’t handle all this pressure … The most convenient way to forget all my problems and have a good sense of self-esteem is using drugs” (P31; 43 years old, divorced, primary school education, an opium user).

Stressful events that stimulate negative emotions, such as the deaths of relatives, also lead to relapse.

*“I might not have reused heroin after abstinence if my father had not died. He supported me in facing my problems”* (P9; 34 years old, primary school education, married, crack heroin user).

For some participants, failure in romantic relationships was closely linked with their drug use problems.

*“My biggest challenge after five months of recovery is that I cannot accomplish my work and life without love. You know, I was going to die even if [he] returned …*” (P9; 34 years old, primary school education, married, crack heroin user).

Psychological factors (such as temptation, craving, social pressure, stressful events, frustration and hopelessness, failure, feelings of anxiety, and romantic failure) often lead to relapse. Based on participants’ beliefs, it seems that some women with a substance use disorder’ recurrent craving for drugs is likely to be shaped after detoxification; consequently, it is necessary to find suitable treatments to stop such craving. In this regard, psychological and educational services can be supportive.

### Contributing factors from family atmosphere: rejection by family, lack of family support, family conflicts, divorce, neglect from family, addiction in the family, spousal betrayal, and failure to maintain romantic relationships

Most participants said that a lack of family support during times of abstinence impacted their relapse. They spoke about many aspects of alienation, such as living alone, living away from family members, being rejected by family, having family conflicts, being neglected by family, getting divorced, and being betrayed by their husbands. One woman explained the need for family support to prevent her relapse into drug use:

*“Many times I felt that I required support, but I didn’t receive it. I think I want to get support from my family; otherwise, I’m tempted to use drugs again because I feel abandoned”* (P10; 31 years old, divorced, primary school education, an opium user).

A participant also clarified the effect of family conflicts on her frustration in the context of drug use:

*“I want to use [drugs] sometimes when I feel alone, when I am bad tempered due to my family conflicts, and my mood is better when I use [drugs] again...” (P13; 40 years old, married, primary school education, an opium user).*

*“I participated in MMT. But a major barrier was that my family had a negative attitude toward methadone. They think of methadone as an addictive drug like crack or opium … They think I am still an addict...”* (P16; 29 years old, high school education, divorced, an opium user).

The same participant said that the contextual and social challenges she faced compelled her to use illicit drugs:

*“The biggest challenge that I am facing right now in my life is my divorce. I cannot deal with this much stress when I have an upset mind. You know, even when I use, it makes me feel better …*” (P16; 29 years old, high school education, divorced, an opium user).

Some participants explained how they relapse into drug use a short time after going through withdrawal. Participants described an overwhelming urge to use illicit drugs because they felt neglected by their families.

*“If my family had paid more attention to me and supported me [in going] to a clinic for drug withdrawal, perhaps I could have overcome my problems and the effects of drug withdrawal. In that case, I may have quit illicit drugs without suffering much”* (P16; 29 years old, high school education, divorced, opium user).

Participants also believed that the illicit drugs used by some of their family members motivated their own relapse.

*“After [quitting drugs], I lived with my grandmother for a few months. My grandmother used opium. I heated the brazier for my grandmother and took a few puffs. Sometimes, I collected all the remains of the burned opium, and I hid [them] from her. It felt good using this stolen opium”* (P10; 31 years old, divorced, primary school education, an opium user).

One of the participants noted that she relapsed and reused illicit drugs as a result of her husband betraying her.

*“I fell in love with my husband and he loved me, too...after my addiction, and when I went to MMT, my husband betrayed me... For me, everything ended [in] that moment. I tried to reuse illicit drugs by any means...”* (P13; 40 years old, married, primary school education, an opium user).

In sum, relapse of drugs is a significant social and personal problem that is impacted by the family atmosphere, including rejection by family, lack of family support, family conflicts, divorce, family neglect, addiction in the family, and husband betrayal. Still, the family often remains the main source of attachment, support, and socialization for drug users in the process of treatment. Thus, the impact of family members’ support during the treatment process of substance use must be considered.

### Socioeconomic conditions: interaction with other people with a substance use disorder, lake of appropriate treatment, easy access to drugs, rejection from friends, unemployment, poverty, and low cost of drugs

Relapse happens in a context of unfavorable social conditions and poor economic resources. Almost all participants had feelings of being judged negatively by relatives and others in society. The most difficult problem one participant who relapsed faced was living with people with whom she had a history of drug use. Friends who use illicit drugs have also been known to be an excuse for using illicit drugs again. Several participants explicitly mentioned the role of friends’ drug use. One woman stated that her friend influenced her to reuse illicit drugs. She said that having direct relationships with addicted friends is associated with a substantial risk of relapse:

*“I have several close friends. Some of them were involved in drug trafficking and drug deals and almost convinced me to reuse illicit drugs”* (P8; 34 years old, primary school education, single, an opium user).

Almost all women faced financial difficulties, and many women considered their poor financial situations to be accelerating their relapse. Because most of them are unemployed and members of poor families, they do not have enough money to continue treatment, and, as a result, they relapse.

*“Due to the stressful conditions after drug withdrawal and the lack of support, most of us relapse in the first three months. There’s no financial support to get medical supplies or even to buy food”* (P18, 41 years old, divorced, secondary school education, an opium user).

Having financial difficulties and easy access to illicit drugs tempted those without full-time work during withdrawal to relapse.

*“I have no permanent job. I must get money from anyone I can. My boyfriend gives me money instead of selling illicit drugs for him...”* (P10; 31 years old, divorced, primary school education, an opium user).

Some participants noted that the exposure to illicit drugs was the main problem they faced. Furthermore, easy access and the low cost of illicit drugs in their previous living environment were considered to be major challenges to sobriety. For instance, one woman whose drug involvement led to her imprisonment said,

*“It’s totally out of control because of the low cost of illicit drugs and easy access to illicit drugs every single day, on the corners...”* (P18, 41 years old, divorced, secondary school education, an opium user).

Generally, several socioeconomic challenges had a strong influence on these women’s relapse into drug use.

Another participant said,

*“Before becoming clean, I spent hours per day for preparation and consumption... After being clean, I lost my friends. [Furthermore], I had a great amount of time [where] I did not have anything to do. My joblessness motivated me to rethink my drug use”* (P7; 36 years old, divorced, high school education, an opioid user).

Unpleasant social situations and poor economic resources could lead to relapse, which was reported in almost all interviews. Obviously, illicit drug use results in an important socioeconomic problem and has been related to a range of negative consequences such as reduced educational attainment, unemployment, and poverty.

### Cultural factors: stigmatization, misconceptions about recreational drug use, sexual disharmony and power imbalance between treatment provider and client, and discrimination

Stigmatization was especially difficult for women who tried to stay away from illicit drugs. These women mentioned that they were seen as drug-addicted persons, despite their sobriety, and were continuously stigmatized. They believed that it was nearly impossible to hide their previous lives as addicted women. They refused to go to treatment centers because of their fear of being labeled as addicted women who had prostituted themselves to buy illicit drugs. Relatives and even close family members distrusted them and deprived them of any support. For instance, one woman said,

*“Even when I abandoned illicit drugs, others wouldn’t accept it. When I went out into the community, many people stigmatized me. Relatives … they did not look at me … They still felt ashamed to be connected with me. Some didn’t trust me. I felt that I had no close friends at that time. I hate going to the drug treatment center. I feel all the people looking at me as if I am a harlot or prostitute*” (P1; 31 years old, single, an opium user).

The women said that they were suffering from stigmatization. They found it almost impossible to hide their lives as addicted women. Some clinics did not accept them due to the need to prevent future problems. Some were faced with sexual requests from patients at a number of facilities, which created a feeling of insecurity that resulted in a lack of follow-up appointments by women.

*“I went to a clinic for treatment. Unfortunately, I faced several shameless requests: sexual offers. I do not want to go to a camp or a clinic for treatment. These places have nothing more than negative views of me and others like me... They believe that addicted persons have bad character and are valueless in a community. Knowing that I am addicted is an ignominy and gives me a sense of shame* …” (P7; 36 years old, divorced, high school education, an opioid user).

Sexual disharmony between treatment provider and client was also reported by some participants. The women believed that the lack of female-specific treatment services affected their treatment outcomes.

*“I have previously visited a methadone treatment center with a male employee. I felt uncomfortable with him. I felt so ashamed around him. When [he was] talking or laughing with colleagues, I felt [that] he [was mocking] me. That’s why I left methadone treatment. I wish women worked there instead; I feel like treatment would be easier that way” (P19; 50 years old, married, high school education, an opium user).*

Some women reported experiencing discrimination and inequality in the delivery of health care between men and women. From their viewpoint, society’s view of addicted women is different from that of addicted men. The perspective of addicted women is related to relapse.

*“All addiction treatment centers are dedicated to men in this city, except one camp that is specifically for women. I do not [feel] comfortable going to the methadone clinic”* (P10; 31 years old, divorced, primary school education, an opium user).

Substance use disorder and relapse are often treated as a negative issue affected by cultural factors such as stigmatization, sexual disharmony between treatment provider and client, and discrimination. Not only has using specific substances resulted in social dissatisfaction and moral blame, but society has also identified such behaviors as offenses. Therefore, the social processes and institutions that are made to control substance use might, in fact, take part in its continuity.

## Discussion

To the best of our knowledge, this is the first qualitative study to explore the relapse experiences of Iranian women with a substance use disorder.

Based on the personal stories of female drug users regarding their relapses, we identified five themes that contributed to relapse: physical complications of drug withdrawal, the psychological burden to quit, family atmosphere, socioeconomic conditions, and cultural factors.

### Physical complications from drug withdrawal

Our findings indicate that the physical complications of drug withdrawal are the most important factors in the relapse into drug use of women with a substance use disorder. Previous studies confirmed that relapse is common in women [13, 14, 20]. Women are vulnerable to relapse because during the process of leaving drugs, they experience severe withdrawal symptoms [21–23]. As described by Hudson and Stamp [22], withdrawal symptoms tempt women to relapse. It is worthwhile to mention that not only is pain common in people with a substance use disorder, but it is related to addiction treatment outcome [24]. In addition, perceived pain is related to increased methadone dose in patients following MMT for reducing pain complications [25]. Women are more susceptible to pain-based withdrawal [23]. Higher sensitivity to pain among women increases risk of relapse [26]. It has been demonstrated that pain management strategies have a positive effect on treatment outcomes [27]. This fact thus supports the value of pain management intervention to reduce the risk of relapse among women with a substance use disorder [28]. Additionally, for women in the postpartum period, progesterone therapy seems to be associated with a reduction in cocaine relapse [29].

Hudson and Stamp stated that ovarian hormones might contribute to relapse in women. In their study, women were vulnerable to relapse during the late luteal phase of the ovulation cycle [22]. Thus, it is recommended that relapse prevention programs designed for women take this stage of their ovulation cycle into consideration.

### Psychological burden from drug withdrawal

Another important finding of this study is that the psychological burden of drug withdrawal is related to relapse. This finding is consistent with a study conducted by Johnson and colleagues [30], who found that female cocaine users with depression are at more risk of relapse. A significantly higher proportion of psychiatric disorders has been seen in female drug users, and negative feelings, such as comorbid anxiety syndrome, depression, stress disorders, and experience of loneliness predispose female drug users to relapse [31, 32]. Psychiatric disorders might complicate treatment outcome in women [33]. One study revealed that patients with depressive disorders were 55% less likely to continue a treatment program [34]. This result suggests the need to develop psychological assessments that enable a quick diagnosis and immediate treatment for psychiatric disorders in female drug users. The treatment of mental disorders provided by a trained mental health team could improve retention in women with a substance use disorder [35]. Additionally, women who reported experience of physical and sexual abuse in their life were at risk of relapse in the treatment period [13, 36]. Furthermore, teaching women skills that emphasize coping with craving, anger management, relaxation, life skills, and meditation might be useful.

### Contributing factors from family atmosphere

Another influential factor related to relapse in women with a substance use disorder is the family atmosphere. The findings of our study emphasize the importance of the role of family and relatives in the outcome of treatment. Our study revealed that 63% of participants lived with someone, either a family member or a partner, who had drug-related problems.

This finding is in line with those of other studies. Dolan and colleagues reported that 53% of Iranian women with a substance use disorder had a partner with a drug use disorder [9]. In this study, we found that husbands play an important role in relapse and drug use. There is contradictory information in this regard. This association was also reported by other authors who found that living with family members with drug use disorders increases the probability of relapse [37, 38]. However, one study result showed that living with a spouse or partner who uses illicit drugs predicts a reduced probability of relapse [39]. Briggs and colleagues suggested that recovery from drug use in women with a substance use disorder positively contributes to a relationship [40].

Although a previous study demonstrated that male partners play a significant role in women’s drug use and seeking of treatment [41], according to a study by Johnson and colleagues, women with a substance use disorder were disappointed by their partners when seeking treatment [30]. Some studies have shown the reverse, where men encourage and support their partners to seek treatment [40, 42]. Even though Soyez and colleagues found better treatment outcomes in females with more social support [43], women with a substance use disorder would like to be isolated from others because of feelings of stigma [44, 45]. Isolation and lack of social support are related to more risk for relapse among women. This indicates that emphasis should be given to counseling of couples in the development of a relapse prevention program.

### Socioeconomic conditions

Several socioeconomic characteristics have an effect on treatment outcome and relapse. Most of the participants in this study live in undesirable socioeconomic conditions: They have limited levels of education, are unemployed, live in poverty, experience conflicts, and have a history of drug abuse and criminal activity in their families. This finding is consistent with the results of other studies that focused on relapse [21, 46]. In low socioeconomic conditions, women with a substance use disorder face many difficulties in accessing treatment. These limitations make it difficult for women to change their lives. Our findings suggest that interventions should be designed to increase the empowerment of women and to enable them to control their lives.

The findings of previous studies showed that a lack of financial resources is common in Iranian women with a substance use disorder [47, 48]. Several characteristics are related to drug recovery in women and have an impact on treatment outcome and relapse, including attitudes toward treatment [49], negative expectations regarding treatment outcome [50], a lack of access to gender-appropriate treatment [51], treatment costs [52], pregnancy difficulty, and child care [53].

According to our study, over 90% of participants have applied self-abstinence in the past in an attempt to quit illicit drugs. One explanation for this is that Iranian women with a substance use disorder feel uncomfortable seeking treatment because there is a lack of appropriate services for women. Therefore, women with a substance use disorder who seek treatment face problems. Treatment cost seems to be another barrier for women. Some of the participants in this study indicated that they dropped out of the treatment program because they could not pay the costs. In Iran and several other countries, people with a substance use disorder cannot rely on their insurance to cover drug abuse treatment. However, when people with a substance use disorder are unable to afford *drug treatment*, they might drop out of the treatment program and start to use illicit drugs again. Addiction treatment features are related to retention in treatment among women [53]. Studies have shown that there are complementary therapies. Counseling on domestic violence, free services for transportation, and child care services have proven to be effective in the retention of treatment [54, 55].

### Cultural factors

Most of the participants in this study have dropped out of treatment at least one time. They did not want to continue their treatment for several reasons. For instance, feelings of embarrassment and shame and stigmatization are barriers to going to drug treatment centers. Dolan and colleagues stated that stigma is one of the main barriers to seeking treatment in Iranian women with a substance use disorder [9]. This finding is in line with other studies on drug abuse [56–58]. Stigma and social barriers might deter the demand for drug recovery programs [59].

According to our findings, the participants in this study were referred to mixed-gender centers, which employed male therapists, to receive treatment services. Although many studies found no relationship between the gender of the patient and therapist and the outcome of the patient [60, 61], the results of this study suggest that women with a substance use disorder are more comfortable receiving treatment services from female therapists. Messina and colleagues suggested that women with drug disorders were more satisfied when they went to drug treatment centers with *female staff* [62]. Women with a substance use disorder in female-specific treatment have better outcomes than those in mixed-gender treatment [63]. Also gender-specific treatment is related to higher feelings of safety and comfort among women [64]. This is not surprising, considering the fact that women fear experiencing sexual harassment from men. As such, we suggest developing gender-specific drug treatment centers and culturally sensitive approaches to stigma in Iran. In addition, innovation in addiction treatments and applying new methods could result in lower stigma and fewer treatment barriers among women who seek treatment [65].

This study is also subject to some limitations. First, a limitation is that data were obtained from a limited number of women who sought treatment and who were referred to a drug treatment setting, such as medium-term residential centers, MMT centers, and women’s prisons. For that reason, the results cannot be generalized to other groups of patients with drug use disorders. A second limitation, inherent to all qualitative studies, is that the presence of the interviewer might have encouraged the respondents to take social norms into account when answering questions. Pressure to conform to social norms can lead to the underreporting of socially undesirable behavior and to the over reporting of socially desirable behavior. However, in this study, we used a number of techniques to avoid this, including triangulation use of contradictory evidence, respondent validation, and constant comparison. Respondent validation, which allowed participants in this study to read through the data and analyses and to provide feedback on the researchers’ interpretations of their responses, provided the researchers a method to check for inconsistencies and an opportunity to reanalyze the data. Despite these limitations, this study provides rich and in-depth information regarding women with a substance use disorder in an Iranian community.

## Conclusions

The findings of this study highlight that women may face many barriers for addiction treatment, such as stigma, discrimination, a lack of access to treatment services, and financial problems. Our findings are in line with research that revealed that women have physiologically, psychologically, and culturally unique features with regard to the addiction process, treatment, and maintenance of recovery [22]. Given the lack of female staff in treatment centers, having comprehensive treatment programs conducted by female employees seems an opportune way to make addicted women feel more at ease. Furthermore, policymakers should be committed to providing a nonjudgmental social environment to remove or reduce stigma in public communities toward women with drug use disorders, and the development of treatment-based insurance might encourage women to undergo treatment.

## Data Availability

The datasets generated and analyzed during the current study are not publicly available in order to protect the participants’ anonymity but are available from the corresponding author on reasonable request.

## Abbreviations

DSM-V: Diagnostic and Statistical Manual of Mental Disorders, Fifth Edition
HIV: Human immunodeficiency virus
MMT: Methadone Maintenance Therapy
UNODC: United Nations Office on illicit drugs and Crime
